# *In Silico* before *In Vivo:* how to Predict the Heating Efficiency of Magnetic Nanoparticles within the Intracellular Space

**DOI:** 10.1038/srep38733

**Published:** 2016-12-07

**Authors:** Beatriz Sanz, M. Pilar Calatayud, Emilio De Biasi, Enio Lima, Marcelo Vasquez Mansilla, Roberto D. Zysler, M. Ricardo Ibarra, Gerardo F. Goya

**Affiliations:** 1 Instituto de Nanociencia de Aragón (INA), Universidad de Zaragoza, 50018 Zaragoza, Spain; 2 Departamento de Física de la Materia Condensada, Facultad de Ciencias, Universidad de Zaragoza, 50009 Zaragoza, Spain; 3 Centro Atómico Bariloche/CONICET, Bariloche, CP 8400, Argentina

## Abstract

This work aims to demonstrate the need for *in silico* design via numerical simulation to produce optimal Fe_3_O_4_-based magnetic nanoparticles (MNPs) for magnetic hyperthermia by minimizing the impact of intracellular environments on heating efficiency. By including the relevant magnetic parameters, such as magnetic anisotropy and dipolar interactions, into a numerical model, the heating efficiency of *as prepared* colloids was preserved in the intracellular environment, providing the largest *in vitro* specific power absorption (SPA) values yet reported. Dipolar interactions due to intracellular agglomeration, which are included in the simulated SPA, were found to be the main cause of changes in the magnetic relaxation dynamics of MNPs under *in vitro* conditions. These results pave the way for the magnetism-based design of MNPs that can retain their heating efficiency *in vivo*, thereby improving the outcome of clinical hyperthermia experiments.

As any other therapeutic protocol, magnetic fluid hyperthermia (MFH) aims to achieve the maximum therapeutic effect with the minimum concentration of heating agent. This goal requires precise a priori knowledge of the *in vivo* heating capacity of such agents as magnetic nanoparticles (MNPs). The bottom line is that the magnetic and rheological properties of magnetic colloids in the as prepared state are different from those in the intracellular environment, particularly because agglomeration disturbs MNP magnetic relaxation mechanisms and thus affects the heating efficiency. MFH consists of heating a target tissue or cell to a temperature of 43–48 °C^1^,^2^. The heating mechanism is based on the magnetic losses of previously internalized MNPs under the action of an alternating magnetic field (AMF)^3^. Thus far, the magnetic materials most extensively used in clinical and biomedical applications are two phases of iron oxide with a spinel structure, i.e., Fe_3_O_4_ (magnetite) and γ-Fe_2_O_3_ (maghemite). As such, the physical properties and systemic toxicity of these MNPs are well understood. Thus, it is not surprising that these iron oxide MNPs have also been the materials most used for MFH experiments. Although many studies have dealt with the heating mechanisms of these MNPs in colloidal form, only a few have quantitatively examined the mechanisms by which heating efficiency is systematically lower under *in vitro* or *in vivo* conditions (i.e., after cell internalization) and related to physical and rheological parameters considered when designing optimal heating materials for therapeutic applications^4^,^5^,^6^.

The origin of power absorption in single-domain MNPs under an AMF is essentially the dephasing between the magnetic moment of MNPs and the magnetic component of the AMF^7^,^8^. The net magnetic moment of the single-domain MNPs couples to the magnetic field component of the applied AMF, absorbing energy and transforming it into heat by a thermally assisted relaxation process^9^. This mechanism accounts for essentially all the heat generated by single-domain particles at the low frequencies of MFH experiments (i.e., ranging from 10^5^–10^6^ Hz). The physical parameter that measures the heating efficiency is the specific power absorption (SPA), also known as specific loss power (SLP), which is the power absorbed/released per unit mass of a magnetic material. As the SPA must be maximized to use magnetic colloids for MFH applications, the design of the constituent MNPs requires a theoretical model of their power absorption to identify those magnetic parameters (e.g., dipolar interactions, composition, magnetic anisotropy) governing the SPA under a given experimental situation. This theoretical model should also include the mechanisms by which physicochemical parameters (e.g., viscosity, dielectric constant, hydrophobicity) of the media surrounding the MNPs affect magnetic relaxation and thus SPA values^10^.

Two main magnetic relaxation mechanisms determine the dephasing of the magnetic moment during power absorption, i.e., a mechanical process (Brownian relaxation) by which the MNPs rotate with their magnetic moment fixed at a given crystal axis, and a magnetic process (Néel relaxation) by which the magnetic moments fluctuate without MNP rotation. Theoretical approaches for modelling these mechanisms have been discussed^8^,^11^,^12^, and there is presently a consensus^8^ that Néel relaxation is the dominant process when the effective magnetic anisotropy constant K_eff_ of the MNPs is K_eff_ ≲1 × 10^4^ J/m^3^ (as for Fe_3_O_4_) and in moderately viscous environments with viscosities η ≥ 0.4 mPa*s (for pure water at room temperature η = 1.0 mPa*s)^7^,^13^. The effects of both the magnetic and rheological conditions on SPA can be merged into a single parameter, 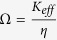, such that in those experimental situations where an 
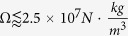
 is obtained, Néel relaxation will be the main contributor to power absorption. For 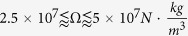 both relaxation mechanisms will contribute simultaneously and, for 
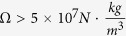
, the Brown relaxation will dominate the heating relaxation mechanism.

Because the ability to generate heat inside cells depends on which relaxation mechanisms^4^,^14^ actually take place within the cell environment (as illustrated in Fig. 1), the challenge of designing intracellular hyperthermia therapy is optimizing the SPA for the dissimilar conditions existing in different tissues and cell types^3^,^15^.

The SPA under *in vitro* (or *in vivo*) conditions varies substantially from that in *as prepared* colloids mostly because when MNPs are taken up by cells, Brown relaxation is drastically stalled, becoming a negligible contribution to heating due to the high viscosity environment, bonding to cell membranes, increased hydrodynamic diameter (e.g., protein corona) and agglomeration. Indeed, a recent work by Soukup *et al*. has elegantly demonstrated that the blocked Brownian relaxation of internalized MNPs can be reversed by lysing the cells^4^. The intracellular viscosity has been reported to be as high as *η* = 50–140 mPa.s^16^,^17^, and since


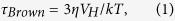


this value alone would account for at least a ≈100-fold increase in the Brown relaxation time compared to the water-based colloid. Hampered MNP free rotation under *in vitro* conditions due to MNP-cell interactions and/or agglomeration^13^ would be plausible explanations for systems with high anisotropy failing to produce large amounts of heat, when the applied magnetic field is not large enough to overcome the anisotropy energy barrier.

Particle agglomeration during cell uptake can also significantly alter Néel relaxation through changes in the anisotropy energy barrier due to dipolar interactions; thus, the validity of the Linear Response Theory (LRT) can no longer be simply extrapolated to predict the SPA under *in vitro* experimental conditions. Previous theoretical and experimental data have demonstrated the influence of particle-particle interactions on the expected SPA^18^ for agglomerated MNPs in the non-linear region (i.e., when the applied field amplitude H_0_ is comparable to the anisotropy field H_A_). These interactions, which can be negligible within protein-rich agglomerates due to surface proteins physically repelling the magnetic cores, can become important in the intracellular space due to the loss of the protein corona, which decreases the average interparticle distance.

This work presents a detailed study of the heating efficiency (i.e., the SPA) of magnetite-based colloids designed to maximize the Néel relaxation under *in vitro* conditions by using a numerical model of SPA mechanisms, including both the observed *in vitro* physicochemical conditions and the effects of H_0_ in the non-LRT region. The magnetic and rheological parameters of the MNPs introduced into the numerical simulation includes (a) dipolar interparticle interactions originating from the agglomeration of MNPs and (b) the viscosity conditions in the intracellular space. Based on these *a priori* considerations, the two types of magnetic colloids prepared to match the requirements (e.g., average particle size, effective magnetic anisotropy) of the constituent MNPs for heating *in vitro* resulted in the largest SPA values yet reported for *in vitro* conditions.

## Results and Discussion

### Physical and Magnetic Characterization

The magnetic colloids synthesized for this work were obtained through an oxidative hydrolysis method modified by the *in situ* addition of a polymer that controls the final particle size and provides a selectable functional surface^19^. Two different polymers, i.e., poly(ethyleneimine) (PEI, M_W_ = 25 kDa) and poly(acrylic acid) (PAA, M_W_ = 450 kDa), were selected to coat MNPs in this work and provide *as prepared* MNPs with different surface charges. The resulting average size of the magnetic cores obtained were <d> = 25 ± 5 nm and 32 ± 6 nm for the PEI-MNPs and PAA-MNPs, respectively. Table S1 and Figure S1 in the supplementary information summarize the basic physical properties of these MNPs. Regarding the magnetic properties, the saturation magnetization M_S_ of PEI-MNPs and PAA-MNPs obtained from SQUID measurements at 300 K were 51 and 54 Am^2^/kg, respectively (Figure S2, Supplementary Information).

**Figure 2:**
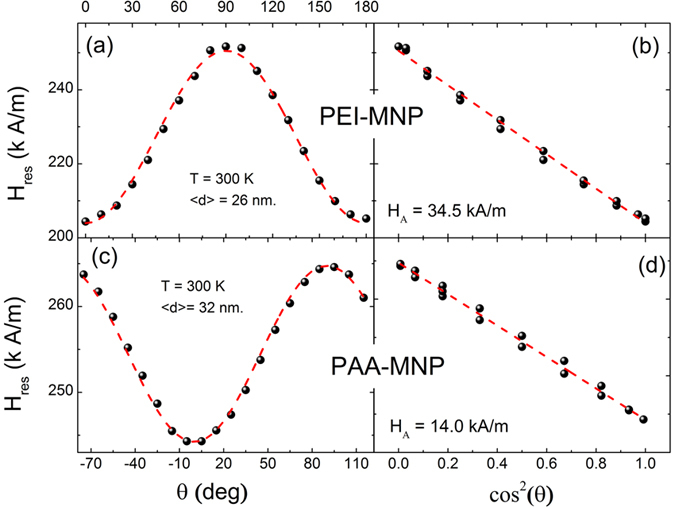
The angular dependence of the resonance field H_res_ for (**a**) PEI-MNPs and (**c**) PEI-MNPs. The linear fit of **H**_**res**_ versus **cos**^**2**^**(θ)** for (**b**) PEI-MNPs and (**d**) PAA-MNPs.

The anisotropy field H_A_ was determined from direct ferromagnetic resonance (FMR) measurements (Fig. 2) through the angular dependence of the resonance field H_res_ in oriented samples^20^. The upper panels (a and b) of Fig. 2 correspond to the PEI-MNPs having an average core size of 26 nm, and the lower panels (c and d) show the corresponding data for the PAA-MNPs with an average size of 32 nm. The data from Fig. 2 show an evident uniaxial symmetry of the magnetic anisotropy, demonstrated by the linear dependence of H_res_ with cos^2^(θ) (right panels, b and d), where θ is the orientation of the sample with respect to the applied field. We recall here that FMR measurements were performed on oriented samples (drying under an external magnetic field of H = 636.6 kA/m),

Although the anisotropy fields H_A_ of both types of MNPs revealed the same uniaxial symmetry, the larger anisotropy found for PEI-MNPs (H_A_ = 34.5 kA/m) than for PAA-MNPs (H_A_ = 14 kA/m) is probably associated to the high fields used in FMR measurements (H ≈ 250 kA/m), which are much larger than those used in SPA experiments (H_0_ ≤ 24 kA/m). These larger fields under FMR experimental conditions make small size differences between samples relevant regarding internal magnetic modes during magnetization reversion, yielding different effective magnetic anisotropies.

### Hyperthermia Experiments

**Figure 3:**
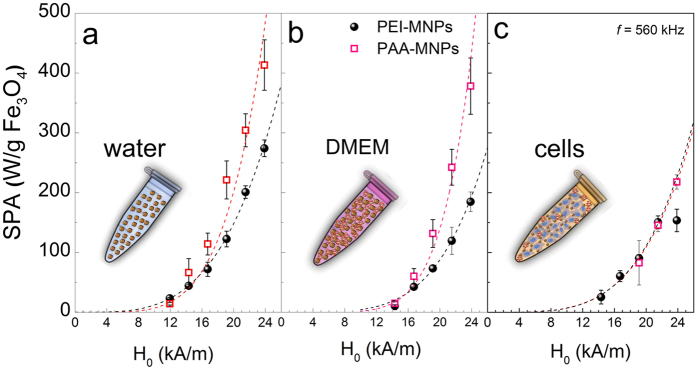
SPA(H) dependence on magnetic field H0 for PEI-MNPs (black solid circles) and PAA-MNPs (red open squares) in water, culture medium (DMEM) and in the cell environment after uptake. Experimental data points were obtained using a commercial device. Dashed lines represent the best-fit curves, as determined by the power equation 
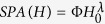
 (see text).

The power absorbed by an ensemble of monodisperse, noninteracting MNPs is expected to follow the relation


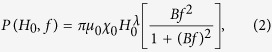


where χ_0_ is the initial zero field equilibrium susceptibility; B = 2π*τ* and λ  = 2 if the LRT conditions are satisfied^8^,^12^. Figure 3 shows the SPA(H_0_) dependence of both colloids *as prepared* in water as a function of applied field amplitude from H_0_ = 12 to 23.9 kA/m at fixed *f* = 560 kHz. The SPA experimental data were fitted with the power law from equation (2) (SPA = Φ·H_0_^λ^) with Φ and λ as free parameters (dashed lines in Fig. 3). As discussed in the introduction, the Ω = K_eff_/η values derived from H_A_ and *η*_*H*2*O*_ of these colloids imply that Néel relaxation should be the dominant process. We recall that the H_A_ and H_0_ values of our experiments invalidate the LRT approximation (i.e., a value of λ = 2), at least for the largest H_0_ amplitudes used. Accordingly, Fig. 3a shows that the H-dependence of the SPA for both types of MNPs deviates from an SPA ∝ H^2^ function and follows an H^λ^ function, with λ = 3.6 ± 0.1 and λ = 4.8 ± 0.3 for PEI-MNPs and PAA-MNPs, respectively. The λ > 2 values can be understood because approaching the H_0_ ≈ H_A_ condition means that the area under the M-H hysteresis loop increases more quickly and in a non-linear manner, resulting in the λ values observed for the SPA.

Figure 3b shows the dependence of the SPA(H_0_) data (*f* = 560 kHz) for MNPs dispersed in the Dulbecco’s modified Eagle’s medium (DMEM) used in our cell cultures. When compared to the SPA values of *as prepared* colloids in water, the values observed for the same MNPs dispersed in DMEM were systematically lower. As the viscosity η of both media are quite similar (i.e., η_DMEM_ = 0.98 mPa.s and η_water_ = 1.00 mPa.s at T = 20 °C), the Brownian relaxation given by equation (1) should not be affected. The time evolution of the hydrodynamic radius of MNPs in cell culture is a complex process that is strongly dependent on the surface characteristics of the particles^21^. In any case, the large increase in the hydrodynamic radius of the MNPs resulting from the formation of a protein corona and agglomeration (Table S1, Supplementary Information) yields hydrodynamic volumes (V_H_) for which the Brown relaxation no longer contributes to the total SPA.

Using the same power law used for the *as prepared* colloids, the experimental data of MNPs dispersed in DMEM (Fig. 3b) could be fitted with the λ values 4.3 ± 0.3 (PEI-MNPs) and 6.2 ± 0.4 (PAA-MNPs), which are 20–30% larger than the corresponding found for MNPs in water. This effect can be understood by the steep increase of the M(H) cycles that occurs when approaching the H_0_ ≈ H_A_ condition discussed above; this effect is related to the Néel contribution and would therefore be more pronounced when Brownian relaxation is absent. The physicochemical characterization of both colloids in water and DMEM indicates that in these conditions, magnetic interactions are not relevant because the *as prepared* MNPs in water showed hydrodynamic radii compatible with a good dispersion, while the protein corona that forms in DMEM minimizes dipolar interactions by separating the MNPs.

The SPA(*f*) dependence of both MNPs at fixed H_0_ = 18.5 kA/m showed the same general characteristics as the field dependence, i.e., smaller SPA values when dispersed in DMEM due to the lack of Brownian relaxation. Because the factor 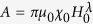 in equation (2) is constant at a fixed H_0_, a 
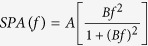
 dependence is expected, in agreement with the non-linear dependence of SPA(*f*) (at H_0_ = 23.9 kA/m) observed from the experimental data (Figure S3, Supplementary Information).

These non-linear effects with H_0_ and *f* discussed above are relevant if the heating response of a colloid is to be extrapolated to the calculation of thermal doses for clinical MFH applications. Moreover, as will be shown below, the effectiveness with which MNPs generate heat under *in vitro/in vivo* conditions can also be affected by dipolar interactions.

**Figure 4:**
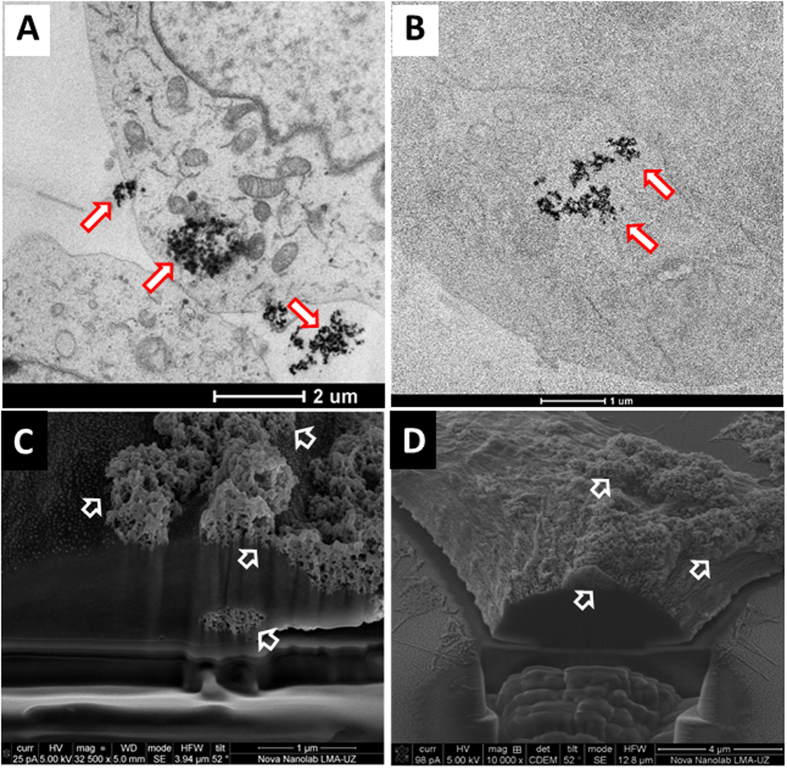
TEM (**A,B**) and FIB-SEM (**C,D**) images of SH-SY5Y cells incubated with PEI-MNPs and PAA-MNPs for 24 hours. The TEM images of (**A**) PEI-MNPs and (**B**) PAA-MNPs show that the internalized particles formed clusters. The FIB-SEM images of the same cells show the presence of dense agglomerates of (**C**) PEI-MNPs and (**D**) PAA-MNPs attached to and crossing the cell membrane.

Prior to determining the SPA (H, *f*) dependence *in vitro*, the cellular uptake and distribution of the particles were analysed to assess possible differences between the final distributions of positively charged PEI-MNPs and negatively charged PAA-MNPs. The images obtained from transmission electron microscopy (TEM) and focused ion beam scanning electron microscopy (FIB-SEM) showed as a general trend that both MNP types aggregate in large clusters localized within the cytoplasm, as expected (arrows in Fig. 4A and B). A significant fraction of the incubated MNPs were also observed to be attached to the cell membrane (arrows in Fig. 4C and D), and cross-sectional images demonstrated that these agglomerates often extended across the cell membrane into the cytoplasm (see also Figures S4–S6, Supplementary Information). No significant numbers of particles were found within endosomes, as previously described elsewhere^21^. The location where MNPs form dense aggregates is relevant for understanding the SPA *in vitro* because these aggregates indicate that magnetic interactions between particles must be included in any model and/or numerical simulation.

After cell uptake, the SPA of both types of MNPs showed a pronounced drop (Fig. 3C) compared to the *as prepared* and DMEM-dispersed data. Notwithstanding the smaller SPA values, the values for λ obtained using the same power law were 4.0 ± 0.3 (PEI-MNPs) and 3.9 ± 0.3 (PAA-MNPs), equivalent to the values observed in water and DMEM, within experimental error. Both the lower SPA values and the similar H^λ^ dependence for the MNPs after uptake are explained by the final intracellular distribution discussed above; the observed compact agglomerates suggest that the protein corona is lost during uptake, increasing the dipolar interactions between particles.

**Figure 5:**
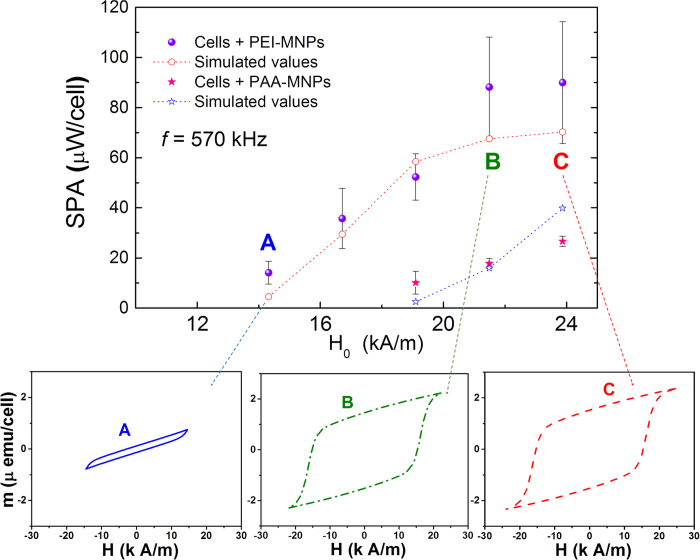
Experimental values of SPA (μW/cell) of MNP-loaded cells as a function of field amplitude (f = 560 kHz). PEI-MNP (filled circles) and PAA-MNP (filled stars) values are compared to simulated SPA values (open symbols). Lower panels display simulated hysteresis loops at selected H_0_ values (points **A**, **B** and **C**).

It is worth noting that the SPA values of the PEI- and PAA-MNPs observed after cell uptake were similar (Fig. 3C), despite differences in the way cells uptake negatively and positively charged MNPs. To evidence these differences, the normalized SPA values per cell (in μW/cell) are shown in Fig. 5 as a function of H_0_ (at *f* = 560 kHz).

The analysis of the SPA per cell (μW/cell) allows the inclusion of the impact of cell uptake for a given MNP type on the final heating efficiency. We note that the experiments were designed to have similar numbers of cells within the pellets because, as already discussed, heat dissipation depends critically on sample volume. The SPA of both types of MNPs was sufficient to not only reach the hyperthermic temperature range in this tumour-like environment (i.e., cell pellets having 7 × 10^6^ cells in a volume of 100 μL) but also induce controlled amounts of cell death. The SPA in a single cell was higher with both particles than previously reported values^22^,^23^. In high fields, the SPA of the PEI-MNPs becomes saturated at approximately 65 μW/cell, which was not observed for the PAA-MNPs, despite the latter having a smaller SPA value.

To interpret these results, we performed numerical simulations considering an interacting nanoparticle system under the action of an AMF with amplitude H_0_. From the FMR measurements we estimated the effective anisotropy field values for the two samples in aqueous solution, and these values were used as input for the numerical simulations, to reproduce the experimental SPA of *as prepared* and *in vitro* conditions. The numerical values obtained were similar to those obtained from FMR measurements, and were between those values expected for the magneto-crystalline anisotropy of bulk maghemite and the magnetite. These results together with the size of the MNPs indicate that the anisotropy field does not change significantly. Nevertheless, an increment of the magnetic interparticle interaction energy is expected from the agglomeration. A dipolar field between two nanoparticle very close (as evidenced from TEM images) was estimated to be about 900 Oe, indicating that a random local orientation of individual magnetic moments and specific topographic details of MNPs within a cluster do not inevitably imply a reduced value for the effective interaction field. The calculations are based on a model we previously proposed^18^ that incorporates the dipolar field interaction into the SPA calculations through a mean field approximation. The simulations were performed using the expression for the magnetization M:


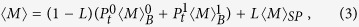


where the brackets indicate the thermal statistical average of the magnetization in the superparamagnetic (SP) and blocked (B) regimes. *L* is the probability to find the particle in the superparamagnetic regime and indicates the population in the *i-th* minimum. The evolution of the magnetization was computed considering that the field H_0_ sweeps changing the probability *L* and the thermal statistical average of M. The input parameters were: H_a_, M_S_, the magnetic diameter (core size), the intrinsic relaxation time, and the parameters associated to the dipolar interaction between the particles. The random orientation for the easy axis of the particles was also considered in our calculations.

This approach considers idealized agglomerates in a hexagonal-close-packed (hcp) arrangement of equal spheres and calculates the magnetic energy *E*_*i*_ for the *i-th* particle located at the centre of the hcp structure from its 12 nearest neighbours. It is worth noting that for a perfectly ordered hcp configuration, the magnetostatic energy can be shown to be *E*_*i*_ = 0, but any real set of spheres with a size distribution will produce distortions in the hcp packing and thus measurable contributions to *E*_*i*_. All simulations were performed at room temperature (T = 300 K) with an intrinsic relaxation time of τ_0_ = 10^−9^–10^−10^ s at different field amplitudes H_0_ but a fixed frequency *f* = 560 kHz to match our experimental conditions. The magnetic diameters of the MNPs used in the calculations were the experimental diameters <d> = 25 ± 5 nm and 32 ± 6 nm (Table S1, Supplementary Information) obtained from the TEM analysis. The dipolar fields were used as free parameters, and the anisotropy field H_A_ values obtained from the FMR experiments for the two types of MNPs were used as initial values for the calculations. The comparison of the measured and calculated SPA values is shown in Fig. 5. The open dots connected by dashed lines are the simulated values, and the right panels show the simulated hysteresis loops of selected data, labelled A, B and C, in the main graph. From these simulations, we found that the interaction fields H_A_ in the mean field approximation were from 2–5% of the corresponding H_0_ fields in the PEI-MNPs and PAA-MNPs. We think that this result is a consequence of the particle packing (we assumed an interparticle distance of ≈5 nm as consequence of the loss of polymeric coating on each particle). Our simulations accurately reproduced the experimental SPA values and confirmed that the relevant relaxation mechanism is the magnetic process. In turn, the lack of Brownian relaxation agrees with the agglomeration of the MNPs observed both at the cell membrane and in the cytoplasm, as illustrated in Fig. 4 (Figures S4–S6, Supplementary Information).

The saturation of the SPA values observed for PEI-MNPs at high H_0_ values is due to the fact that somewhere from H_0_ = 22–24 kA/m, the hysteresis loop at that frequency reaches the irreversibility point, i.e., the M vs. H curves correspond to a major hysteresis loop. The lower panels of Fig. 5 illustrate this behaviour. Importantly, the values used in the simulations for the anisotropy fields H_A_ were those obtained from the FMR data for the PEI-MNPs. In the case of PAA-MNPs, a value of H_A_ = 55 kA/m was needed to obtain good agreement with the SPA vs. H_0_ data. This value is larger than the H_A_ = 14 kA/m measured via FMR, and this difference is likely due to the different field regimes used in the two experiments; while H_0_ ≈ 260 kA/m for the FMR experiments, H_0_ ≤ 24 kA/m for the SPA experiments. Thus, in the SPA experiments, the reversion of the magnetization in PAA-MNPs was coherent because H_0_ was much lower than H_A_. In the FMR experiments, the magnetization followed the magnetic field H_0_ easily by the above-mentioned internal magnetic modes to reverse the magnetization. Many studies have aimed to maximize the SPA as the key parameter for quantifying the efficiency with which MNPs convert absorbed energy into heat^24^,^25^,^26^,^27^,^28^,^29^. However, the SPA is not an intrinsic property of the MNPs or the magnetic colloid; instead, the SPA depends on the applied frequency *f* and amplitude H_0_ of the applied magnetic field in a non-trivial way. Moreover, the effect of the viscosity of the liquid carrier must be considered when comparing the SPA of different MNPs. As such, the literature is plagued with considerable confusion on the actual heating performance of MNPs. Indeed, widely different SPA values have been reported, ranging from 10 to 10^3^ W/g^25^,^30^,^31^,^32^, depending on the magnetic field conditions used (4 < H_0_ < 100 kA/m and 10 < *f* < 5 × 10^4^ kHz). In addition, most of the largest values correspond to the *as prepared* colloids because the contribution of Brown relaxation in a lower viscosity environment adds to the total power absorption. In contrast, a major limitation of heating small tissue volumes or single cells is the lack of sufficient MNP heating efficiency with clinically acceptable values of MNP concentration, field amplitude and frequency. Some attempts to circumvent this limitation have used dangerously high frequencies and/or larger quantities of particles^33^,^34^. Table 1 shows a compilation of previously reported *in vitro* SPA measurements. Although the SPA values of the *as prepared* colloids in this work are comparable to previously reported values^24^,^35^, our *in silico* design of MNPs based on theoretical simulations allowed the MNPs to retain their SPA under *in vitro* conditions. Indeed, to the best of our knowledge, these SPA values are the largest values reported under similar (H, *f*) conditions, as displayed in Table 1, making them the most effective *in vitro* nanoheaters yet reported^36^,^37^. Our findings clearly support the notion that for the adequate use of MNPs as nanoheaters *in vitro* or *in vivo*, it is crucial to engineer the MNPs based on the subtle interplay of the magnetic anisotropy, dipolar interactions and viscosity of the environment surrounding the MNPs.

Our model and the calculations used here are expected to apply for a) single-domain MNPs and b) within the experimental timeframe of hyperthermia measurements. A different, promising approach using multi-core MNPs composed of single-domain units densely packaged into a MNPs nucleus, has been reported by Dennis *et al*.^38^ For these MNPs the interparticle interactions should play a major role, and the large and saturation magnetization reported suggest that exchange interactions across boundaries are active to keep the magnetic moments aligned. This possibility of switching on exchange interactions could open new ways to increase the SPA through materials engineering.

In any case, as the observed intracellular heating is often due to the Néel relaxation of the magnetic moment through the energy barrier in an interacting system, detailed models of MNPs agglomerates, together with their experimental characterization, will be most helpful for understanding how to control the thermal dose in therapies requiring magnetic hyperthermia.

## Conclusions

Collectively, these results show how two seemingly similar magnetic colloids (in terms of a basic characterization of the magnetic or physicochemical properties) can differ regarding their properties as magnetic nanoheaters for *in vitro* magnetic hyperthermia. Due to the high intracellular viscosity, the magnetic relaxation is the relevant mechanism to be optimized for improving the SPA when nanoparticles are internalized or attached to the cell membrane. Differences between the nanoparticles in the dependence of the SPA on the amplitude of the applied field are mainly related to differences in the anisotropy field and magnetic diameter of the nanoparticles, which, together with the local viscosity and dipolar interaction, determines the behaviour of MNPs within a tumour microenvironment. Therefore, preliminary knowledge must be acquired via numerical simulation before the best candidates for future *in vitro* or *in vivo* magnetic hyperthermia treatments can be chosen. Regarding iron oxide MNPs, their ‘low’ magnetic anisotropy renders the LRT approximation invalid for magnetic fields ≈25 kA/m. The experimental observation of compact MNP aggregates forming after cell uptake pointed to magnetic interparticle interactions as the origin of the lower heating performance *in vitro*, while numerical modelling allowed the impact of these magnetic dipolar interactions on the SPA to be quantified. These magnetic interactions, together with the anisotropy field of MNPs, determine the actual heating efficiency of MNPs for *in vitro* hyperthermia experiments.

## Materials and Methods

### Materials

All reagents were commercially available and used as received without further purification. Iron (II) sulphate heptahydrate (FeSO_4_·7 H_2_O), sodium hydroxide (NaOH), potassium nitrate (KNO_3_), sulfuric acid (H_2_SO_4_), poly(ethylenimine) (PEI, M_W_ = 25 kDa) and poly(acrylic acid) (PAA, Mw = 450 kDa) were obtained from Sigma-Aldrich.

### Synthesis of Magnetic Colloids

Two different magnetic colloids were synthetized via a modified version of the classical oxidative hydrolysis method, i.e., the precipitation of an iron salt (FeSO_4_) in basic media (NaOH) with a mild oxidant^19^. The method was modified to produce an *in situ* polymer coating with PEI of 25 kDa and PAA of 450 kDa during the reaction, yielding PEI-coated MNPs (PEI-MNPs) and PAA-coated MNPs (PAA-MNPs). The synthesis process of both water-based, stable, magnetic ferrofluids containing coated MNPs has been previously described elsewhere^21^.

Both water-based, stable, magnetic ferrofluids containing coated MNPs were studied to determine various properties, such as the surface charge, the resistance to aggregation and the number of available functional groups on the particle surface, with respect to each polymer^21^. Both magnetic colloids were similar in average size, which ranged from ≈25–32 nm with a narrow size distribution. The characterization of the properties of MNPs under different conditions (i.e., dispersion media) is decisive for the success of specific applications. The ability of MNPs to adsorb proteins is expected to depend on the physicochemical characteristics and the surface coating; i.e., its affinity for the adsorption of ions, proteins and natural organic materials. The physiological medium will influence nanoparticle properties, such as size, aggregate state, morphology, surface charge, surface composition and magnetic response. Thus, these properties were studied in water and in Dulbecco’s modified Eagle’s medium (DMEM, Lonza S.L., Porriño, Spain) supplemented with foetal bovine serum (15% FBS).

### Cell Culture

Human SH-SY5Y neuroblasts (ATCC CRL-2266) were cultured in DMEM and Ham’s F12 (1:1) with 15% FBS, 100 IU/mL penicillin, 100 μg/mL streptomycin and 2 mM L-glutamine. Cells were maintained at 37 °C in a saturated humid atmosphere containing 95% air and 5% CO_2_. SH-SY5Y cells were incubated with 100 μg/mL of both nanoparticles (PEI-MNPs and PAA-MNPs) overnight. After this incubation, the cells were washed, and the modified DMEM was replaced with ordinary DMEM. Control experiments were performed with growth medium without nanoparticles.

### Determination of Iron Content in the Magnetic Colloids and Intracellular Space

The concentration of ferrite nanoparticles in both magnetic colloids was determined from their Fe content by VIS-UV transmission spectrophotometry (Shimadzu UV-160). The protocol is based on the thiocyanate complexation reaction^39^:


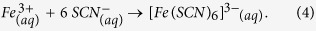


For this determination, the PEI-MNPs and PAA-MNPs were dissolved in HCl 6 M HNO_3_ (65%) at 50–60 °C for 2 h. Potassium thiocyanate was then added to the Fe^3+^ solution to form the iron-thiocyanate complex, which has strong absorbance at a wavelength of 478 nm.

### Specific Power Absorption Measurements

The specific power absorption (SPA) experiments in different dispersion media (e.g., water, culture media and the intracellular space) were performed in a commercial magnetic field applicator (DM1 applicator, nB Nanoscale Biomagnetics, Spain) using magnetic fields from 3.98 to 23.9 kA/m at several frequencies, ranging from 260 to 832 kHz. Calorimetric experiments were conducted by exposing 1 mL of the magnetic colloid in a vacuum-insulated Dewar connected to a vacuum pump (10^−7^ mbar). A fibre-optic measuring probe was placed at the centre of the colloid to sense the temperature of the sample. A ‘dead time’ of 3–4 minutes was allowed before each experiment for the samples to reach thermal equilibrium. We approximated the measurement as being adiabatic, i.e., neglecting the heat losses through the container walls. Under this hypothesis, the SPA equation can be written as


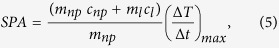


where the parameters *m* and *c* are the mass and specific heat, and the subindex *np* and *l* indicate nanoparticles and liquid, respectively. The SPA value is extracted from the slope of the temperature vs. time curves during the initial t < 30 s interval. This linear fit gives the maximum heating rate (ΔT/Δt)_max_ of the sample [K·s^−1^]. The error was estimated from the standard deviation of the number N of measurements for each (H_0_, *f*) set of values (N > 3 in all cases).

### Transmission Electron Microscopy

The distribution and morphology of SH-SY5Y cells incubated with both types of MNPs were analysed by transmission electron microscopy (TEM) using an FEI Tecnai T20 microscope operating at 200 keV. Cell samples were incubated overnight with PEI-MNPs and PAA-MNPs (10 μg/mL). After this incubation, the cells were detached and fixed with 2% glutaraldehyde solution for 2 h at 4 °C, washed three times in cacodylate buffer (pH 7.2), and then treated with potassium ferrocyanide 2.5% and osmium tetroxide 1% for 1 hour at room temperature. After being washed, the cells were dehydrated with increasing concentrations of acetone, i.e., 30% (x2), 50% (x2), 70% (x2), 90% (x2), and 100%. After drying, the samples were embedded in a solution (50:50) of epoxy resin and acetone (100%) overnight and then in 100% epoxy resin for 4–5 h. The sample were dried for 2 days at 60 °C and then cut into 70-nm-thick slices.

### Dual-Beam FIB-SEM Analysis

The intracellular distribution of MNPs in conditioned SH-SY5Y neuroblast samples was studied using dual-beam FIB-SEM (Nova 200 NanoLab, FEI Company). SEM images were taken at 5 and 30 kV with a field emission gun column, and a combined Ga-based 30 kV (10 pA) ion beam was used to view single-cell cross-sections. These investigations were completed by energy-dispersive X-ray spectroscopy for the chemical analysis.

### Magnetization Measurements

For the magnetization measurements, 1 mg of each sample was dispersed in 100 μL of ethanol and added to an epoxy resin to immobilize the nanoparticles and minimize interparticle interactions during the magnetization measurements with an applied field. The final samples used for the magnetic measurements contained approximately 1% *wt.* MNPs. The magnetization measurements were made using a commercial VSM magnetometer at 300 K, and the signal was normalized by the mass of material used.

### Ferromagnetic Resonance Measurements

Samples used for the ferromagnetic resonance (FMR) measurements were conditioned in a similar manner as described for the magnetic measurements. However, the hardening of the epoxy resin (about 24 hours) was performed under an applied field of 636.6 kA/m to mechanically orient the easy axis of the nanoparticles. The X-band (9.4 GHz) FMR measurements were performed using a Bruker ESP-300 spectrometer at 300 K. The spectra were measured by varying the orientation of each sample with a goniometer with respect to the applied field; θ is the angle between the field cooling direction and the applied field.

## Additional Information

**How to cite this article**: Sanz, B. *et al*. *In Silico* before *In Vivo*: how to Predict the Heating Efficiency of Magnetic Nanoparticles within the Intracellular Space. *Sci. Rep.* **6**, 38733; doi: 10.1038/srep38733 (2016).

**Publisher's note:** Springer Nature remains neutral with regard to jurisdictional claims in published maps and institutional affiliations.

## Supplementary Material

Supplementary Information

## Figures and Tables

**Figure 1:**
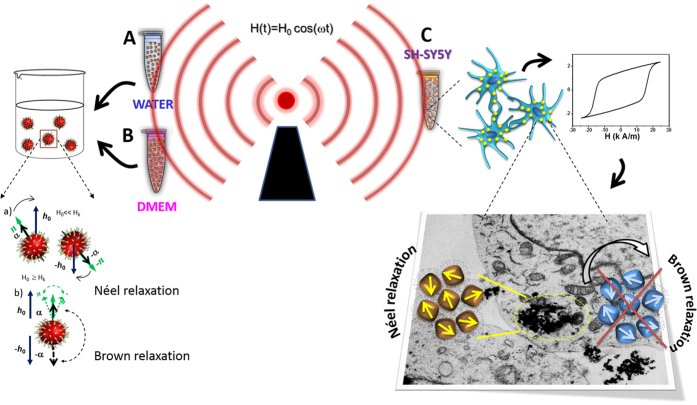
Schematic representation of the experimental approach for determining the contributions of relaxation mechanisms, i.e., Néel relaxation (τ_N_) and Brownian rotation (τ_B_), in single-domain particles in water (**A**), culture medium (**B**) and cytoplasm (**C**).

**Table 1:** Experimental values of specific power absorption (SPA) reported under *in vitro* conditions in different cell lines.

Medium	Cell line	SPA (W/g)	H_0_ (kA/m)	*f* (kHz)	Reference
	HUVEC	114 ± 21	23.1‡	700	40
	U87-MG	178 ± 37	23.1‡	700	40
	PC3	~180	24.6	1000	36
PBS	—	153.4	10	425	41
Agar	—	≈100	23.9	700	42
Glycerol	—	≈100–130	24.8	700	43
	SH-SY5Y	217.5	24.0	560	This work

^‡^The value H_0_ = 34.4 Oe (2.74 kA/m) was converted to 23.10 kA/m (i.e., 290 Oe) in ref. 37. From previous works of the same group, we chose the more realistic H_0_ = 23.1 kA/m value.
