# Supplementary material for: In Silico before In Vivo: how to Predict the Heating Efficiency of Magnetic Nanoparticles within the Intracellular Space
Source: Sci Rep. 2016 Dec 7;6:38733. doi: 10.1038/srep38733 (PMC5141417; doi:10.1038/srep38733)
Supplement: Supplementary Information [file srep38733-s1.pdf]

Electronic Supplementary Information

Title: *In Silico* before *In Vivo*: how to Predict the Heating Efficiency of Magnetic Nanoparticles within the Intracellular Space

Beatriz Sanz[a,†], M. Pilar Calatayud[a,b], Emilio De Biasi[c], Enio Lima Jr.[c], Marcelo Vasquez Mansilla[c], Roberto D. Zysler[c], M. Ricardo Ibarra[a,b] and Gerardo F. Goya[a,b,*]

[a] Instituto de Nanociencia de Aragón (INA), Universidad de Zaragoza, 50018 Zaragoza, Spain

[b] Departamento de Física de la Materia Condensada, Facultad de Ciencias, Universidad de Zaragoza, 50009 Zaragoza, Spain

[c] Centro Atómico Bariloche / CONICET, Bariloche, CP 8400, Argentina

*Corresponding author

E-mail: goya@unizar.es

[†] Currently at *nB nanoScale Biomagnetics S. L., Zaragoza, Spain.*

Table S1. Saturation magnetization M_S, coercive field H_C, average particle core diameter $<d>$, average hydrodynamic diameter in water $<d_H>_{water}$, and in DMEM $<d>_{DMEM}$, of the magnetic nanoparticles used in this work.

Sample	M_s Am^2/kg	H_c kA/m	$<d>$ nm	$<d_H>_{water}$ nm	$<d_H>_{DMEM}$ nm
**PEI-MNPs**	51.9	5.6	25±5	73±25	3094±730
**PAA-MNPs**	51.6	5.6	32±6	155±25	1305±381

Table S2. SPA values (H_0 = 24 kA/m, *f* = 560 kHz) of *as prepared* magnetic nanoparticles in water, protein-rich medium (DMEM) and *in vitro*. Also de values of exponent λ obtained from the fit of the power law SPA(H) = Φ H^{λ} are tabulated.

**Sample**	**WATER**		**DMEM**		**Cells**	
	SPA (W/g_{Fe3O4})	λ	SPA (W/g_{Fe3O4})	λ	SPA (W/g_{Fe3O4})	λ
**PEI-MNPs**	274±37	3.6(3)	185±16	4.3(3)	157±19	4.0(3)
**PAA-MNPs**	413±42	4.8(3)	378±47	6.2(4)	217±12	3.9(3)

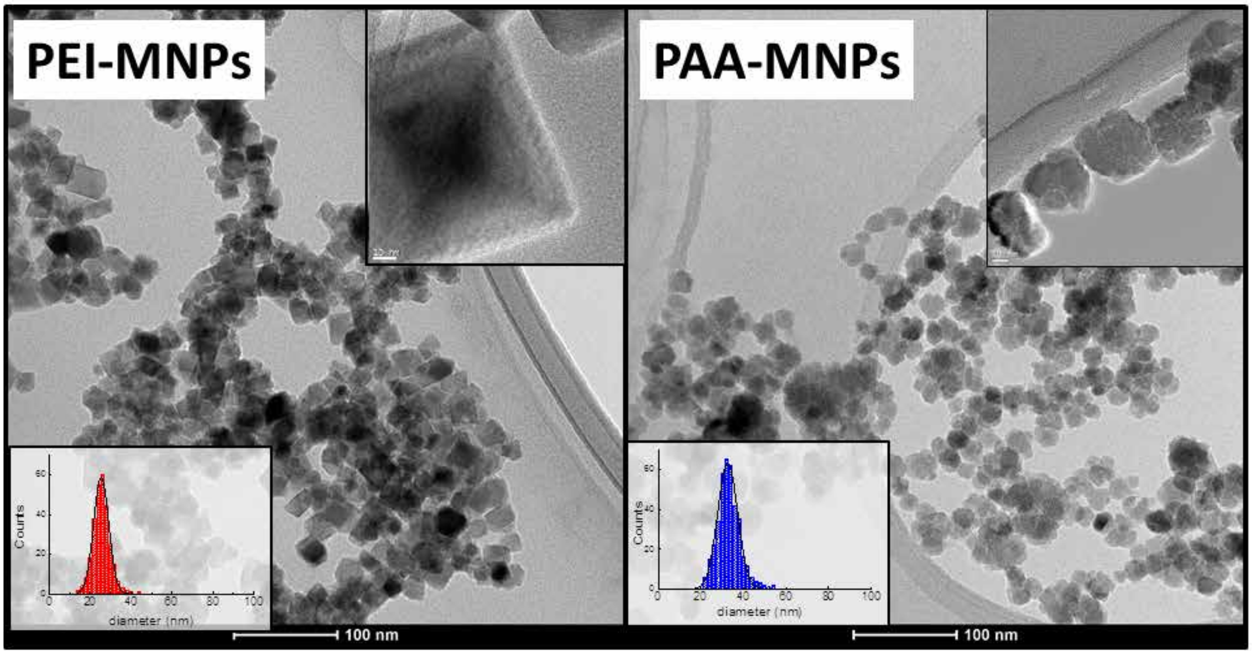

Figure S1. TEM images of the two magnetic particles used in this work. Left panel: PEI-MNPs, magnified area and resulting particle size histogram fitted with a Gaussian function. Right panel: PAA-MNPs, magnified area and resulting particle size histogram.

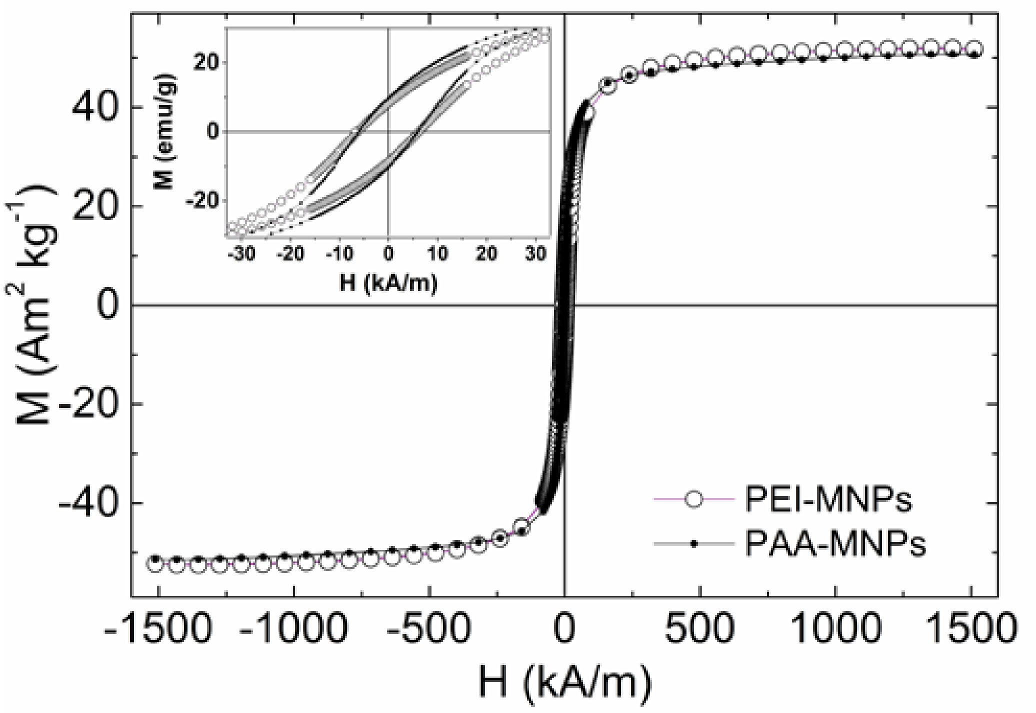

Figure S2. Room-temperature magnetization curves as a function of applied field for poly(ethyleneimine)-coated nanoparticles (open circles) and poly(acrylic acid)-coated nanoparticles (solid circles).

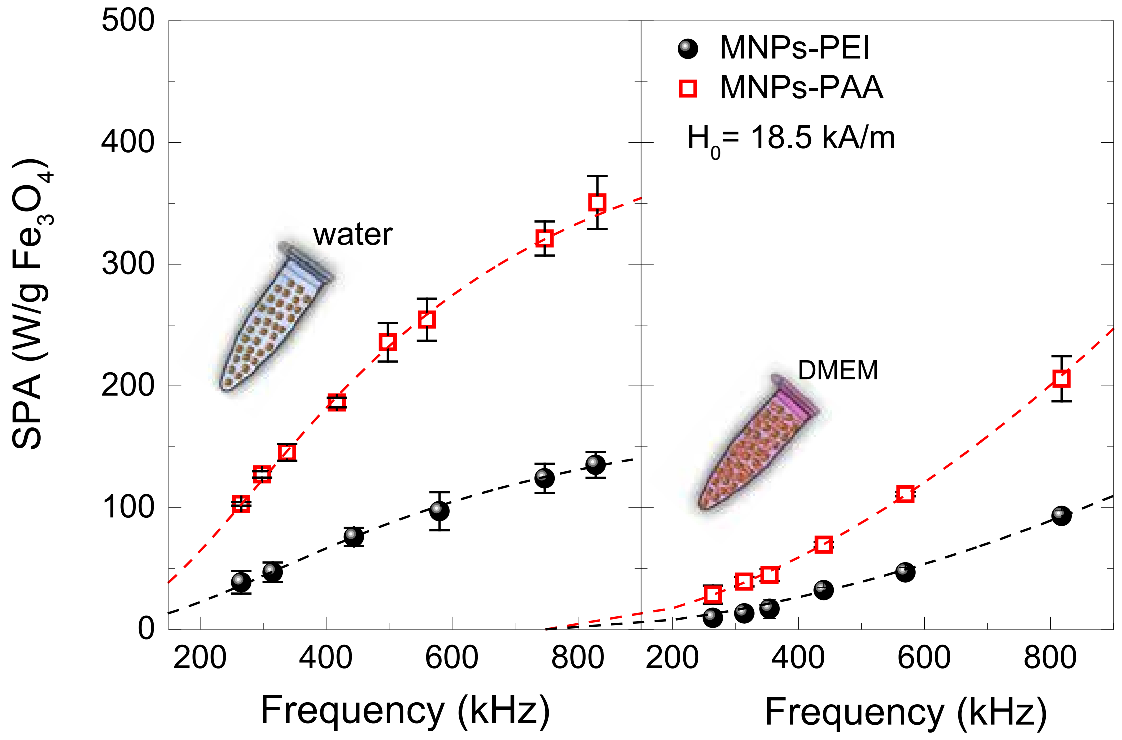

Figure S3. **SPA *vs.* frequency (*f*) measured at a fixed amplitude H_0 = 18.5 kA/m.** Dashed lines are the best-fit curves determined using the relation $\mathrm{SPA(f)} = \mathrm{A}\left(\frac{\mathrm{Bf}^2}{1+(\mathrm{Bf})^2}\right)$.

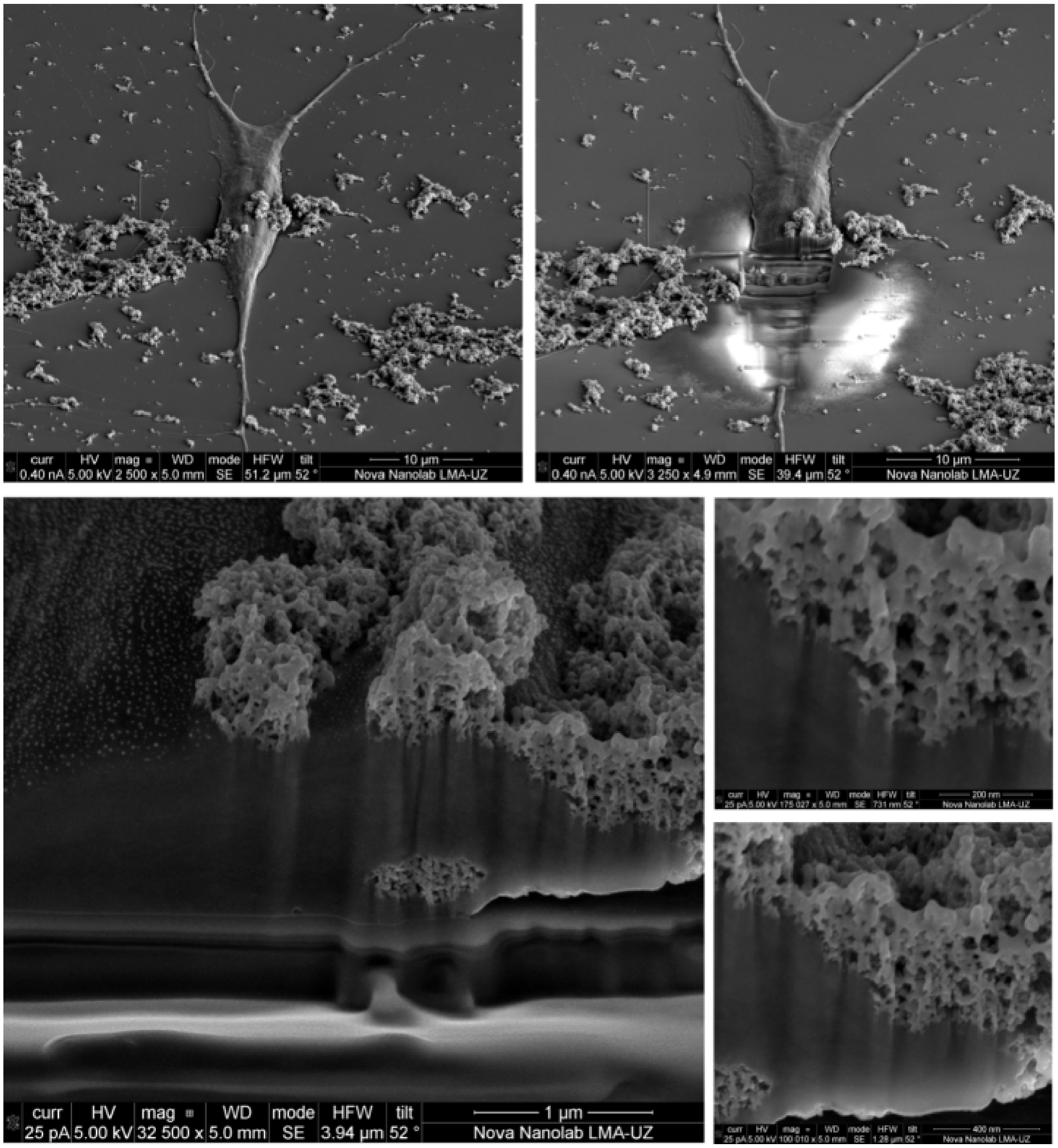

Figure S4. FIB-SEM Dual Beam images of SH-SY5Y cells incubated with PEI-MNPs (100 µg·ml^{-1}) for 24 hours. Lower panels: cross section images showing a detail of the dense cluster structures observed inside the cells and across the cell membrane.

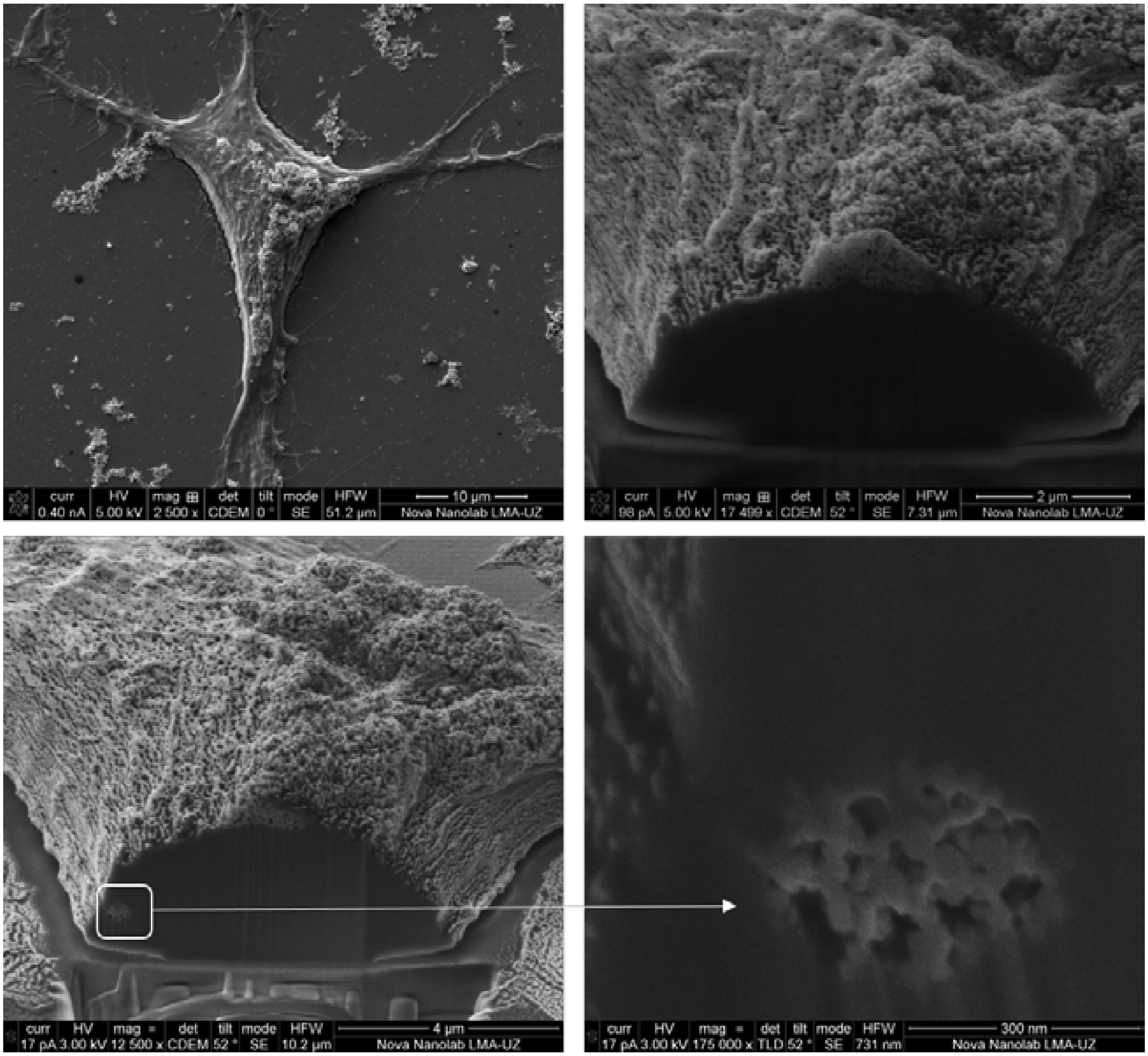

Figure S5. FIB-SEM images of SH-SY5Y cells incubated with PAA-MNPs (100 µg·ml^{-1}) for 24 hours. The lower panels show a cross-sectioned cell (left) and a magnification of the NPs clusters (right)

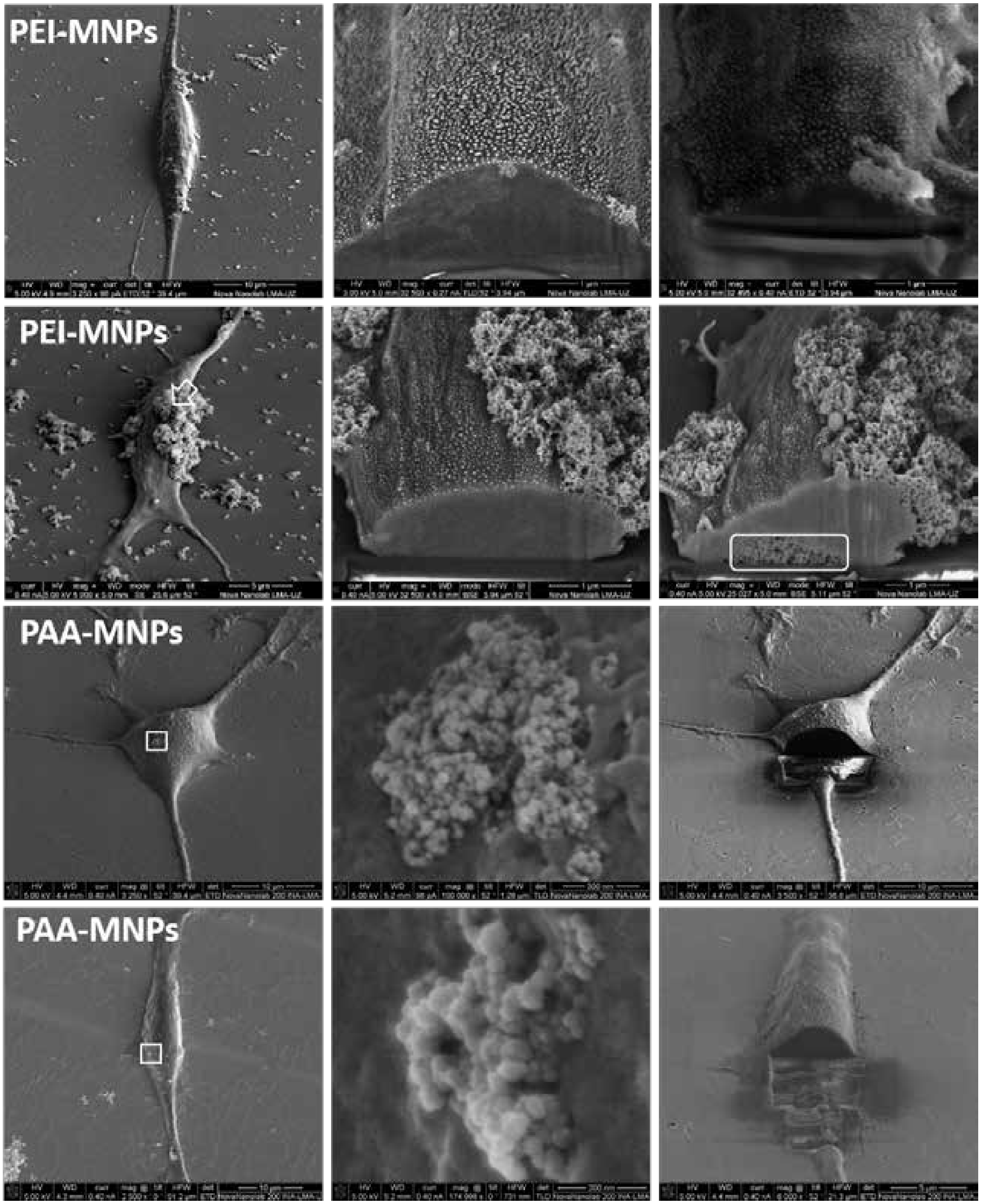

Figure S6. FIB-SEM images of SH-SY5Y cells incubated with PEI-MNPs and PAA-MNPs for 24 hours.
